# PW01-009 – Markers of inflammation in adult FMF patients

**DOI:** 10.1186/1546-0096-11-S1-A62

**Published:** 2013-11-08

**Authors:** A Giese, H Wittkowski, A Örnek, M Kurucay, E Lainka, BF Henning

**Affiliations:** 1 Central Patient Admission Unit / Emergency Department, Marienhospital Herne, Ruhr-University Bochum, Herne, Germany; 2 Department of Paediatrics, University of Münster, Münster, Germany; 3 Department of Paediatrics, University Medical Centre Essen, Germany; 4 AID-NET Autoinflammatory disorders (AID) in children: Genetics, disease mechanisms, diagnostic markers and therapeutic targets, Supported by the German Ministry of Education and Research (BMBF 01GM08104), Essen, Germany

## Introduction

The therapeutic goal in Familial Mediterranean Fever (FMF) is to prevent attacks of clinically overt disease as well as to stop or reverse the development of amyloidosis and subsequent organ damage. The dosage of colchicine treatment is therefore generally adjusted according to clinical information and biochemical markers of subclinical inflammation.

## Objectives

To evaluate whether biochemical markers of inflammation help to differentiate between healthy subjects and FMF patients with or without clinically apparent attacks among adult Turkish migrants living in Germany.

## Methods

40 consecutive patients suffering from FMF according to the Livneh criteria and 40 healthy controls (C) were included into the study in Herne, Germany. All participants were Turkish migrants aged ≥18 years. Patients were excluded if they reported symptoms of FMF ≤7 days prior to inclusion. Frequency of FMF attacks during the 3 months preceding the study inclusion was assessed. Patients were grouped into patients without (F-) or with (F+) attacks. The following markers were determined: erythrocyte sedimentation rate (ESR, ref: <20mm/h), C-reactive protein (CRP, ref: <0.5mg/dl), serum amyloid A (SAA, ref: <0.5mg/dl), fibrinogen (FI, ref: 2.38-4.98 g/l) and S100A12 (ref: <120 ng/ml).

## Results

C (n=40) / F- (n=14) / F+ (n=26) showed the following characteristics (continuous variables are depicted as mean ±standard deviation); age: 34.6±10.7 / 35.3±8.2 / 35.2±11.2 years, female gender: 52.5 / 57.1 / 61.5 %. Age at FMF onset and daily colchicine dose in F- / F+ was 8.7±5.2 / 11.9±7 years and 1.4±0.7 / 1.3±0,9 mg/day. Biochemical markers were as follows for C / F- / F+; ESR: 16.3±12.8 / 26.1±25.1 / 28.7±16.2 mm/h, CRP: 0.35±0.76 / 0.69±0.92 / 0.83±0.97 mg/dl, SAA: 0.92±3.6 / 2.1±3.0 / 2.8±4.8 mg/dl, FI: 2.8±0.5 / 2.9±0.6 / 3.22±0.6 g/l, S100A12 was available only for F- / F+ and amounted to 746±1072 / 3642±9116 ng/ml. C and F+ significantly differed concerning ESR, CRP, SAA and FI but not concerning age and gender. No significant difference could be detected between F- and F+ concerning any of the parameters studied.

## Conclusion

Among Turkish migrants living in Germany FMF patients with ≥1 attack in 3 months (F+) show significantly higher ESR, CRP, SAA and FI compared to healthy controls (C). ESR, CRP, SAA and FI did not permit to differentiate between C and FMF patients without attacks (F-) nor between F- and F+. Between F- and F+ no significant difference in S100A12 levels could be detected.

## Disclosure of interest

None declared

